# Atrial fibrillation in UK South Asian hospitalized ischemic stroke patients: The BRAINS study

**DOI:** 10.1371/journal.pone.0281014

**Published:** 2023-02-07

**Authors:** Taylor Aurelius, Gie Ken-Dror, Sapna D. Sharma, Sageet Amlani, Gunaratnam Gunathilagan, David L. Cohen, Chakravarthi Rajkumar, Stuart Maguire, Sissi Ispoglou, Ibrahim Balogun, Anthea Parry, Lakshmanan Sekaran, Hafiz Syed, Enas Lawrence, Ravneeta Singh, Ahamad Hassan, Chris Wharton, Khalid Javaid, Neetish Goorah, Peter Carr, Eman Abdus Sami, Pankaj Sharma

**Affiliations:** 1 Institute of Cardiovascular Research Royal Holloway, University of London (ICR2UL), London, United Kingdom; 2 BARTS and the London NHS Trust/ Royal London Hospital, London, United Kingdom; 3 Queen Elizabeth the Queen Mother Hospital, Kent, United Kingdom; 4 Northwick Park Hospital, London, United Kingdom; 5 Brighton and Sussex University Hospitals NHS Trust & Brighton and Sussex Medical School, University of Sussex, Sussex, United Kingdom; 6 Bradford Teaching Hospital, West Yorkshire, United Kingdom; 7 City Hospital Birmingham, West Midlands, United Kingdom; 8 William Harvey Hospital, Kent, United Kingdom; 9 Hillingdon Hospital, London, United Kingdom; 10 Luton and Dunstable Hospital, Bedfordshire, United Kingdom; 11 Newham University Hospital, London, United Kingdom; 12 Croydon University Hospital, London, United Kingdom; 13 West Middlesex University, London, United Kingdom; 14 Leeds General Infirmary, West Yorkshire, United Kingdom; 15 New Cross Hospital, West Midlands, United Kingdom; 16 Walsall Manor Hospital, West Midlands, United Kingdom; 17 Queen’s Park Hospital Royal Blackburn, Lancashire, United Kingdom; 18 Birmingham Heartlands Hospital, West Midlands, United Kingdom; 19 Airedale General Hospital, West Yorkshire, United Kingdom; 20 Ashford & St Peter’s NHS Foundation Trust, Surrey, United Kingdom; 21 Department of Clinical Neuroscience, Imperial College Healthcare NHS Trust, London, United Kingdom; Ehime University Graduate School of Medicine, JAPAN

## Abstract

**Introduction:**

South Asian diaspora comprise one of the largest ethnic minority groups in the world yet data about atrial fibrillation (AF) in this demographic is understudied. Our aim is to identify differences in AF prevalence and treatment between South Asians and white British stroke patients.

**Method:**

The UK arm of a prospective ongoing large international repository on stroke was analysed. Ethnic differences in AF prevalence and management in those with ischemic stroke were analysed.

**Results:**

Of the 3515 individuals recruited with ischemic stroke, 1482 (men: 972, women: 510) were South Asian and 2033 (men:1141, women:892) of white British ethnicity. AF was present in 462 white British and 193 South Asians stroke patients, with South Asians displaying a lower prevalence of AF (South Asians: 13.0% vs white British 22.7%, P<0.001). Despite adjustment for traditional AF risk factors, South Asians had a significantly lower OR of AF compared to white British stroke patients (OR: 0.40, 95%CI: 0.33:0.49, P<0.001). Among confirmed AF cases, 31.8% of South Asians and 41.4% of white British were untreated at admission (P = 0.02). Antiplatelet treatment was significantly higher among South Asians at both admission (South Asian: 47.4% vs. white British: 29.9%, P<0.001) and discharge (South Asian: 49.5% vs. white British: 34.7%, P = 0.001), although anticoagulation treatment was similar across both ethnic groups at admission (South Asian: 28.5% vs white British: 28.1%, P = 0.93), and discharge (South Asian: 45.1% vs white British: 43.1%, P = 0.64).

**Conclusion:**

Stroke patients of South Asian descent are at significantly lower risk of AF but more likely to be on antiplatelet treatment compared to their white British counterparts.

## Introduction

Atrial Fibrillation (AF) induces a 5-fold greater risk of ischemic stroke [1] and is estimated to affect 3.3% of the UK population [2] and 1–2% of the US population. Many of the established major risk factors for AF (age, untreated hypertension, diabetes, and coronary heart disease) are themselves linked to increased stroke risk [3].

In the UK, South Asians (Indian, Pakistani, Bangladeshi) make up the largest ethnic minority group, with a population of over 3 million individuals [4,5]. This is comparable to other western countries, with the US reporting ~ 6 million individuals. Despite the higher burden of most primary AF risk factors in South Asians [6,7], prevalence of AF has been reported to be lower compared to white British [8], though these studies have been small and mostly local. Current guidelines recommend the use of anticoagulants as appropriate treatment for AF related stroke prevention, although their use in differing ethnic groups has not been well studied [9].

To investigate differences of AF risk and treatment between UK South Asians and white British ischemic stroke patients we analysed UK data from the ongoing international large prospective BRAINS (Bio-Repository of DNA in Stroke) study.

## Materials and methods

### Data source

The data for this study is sourced from the BRAINS dataset [10]. The UK arm of BRAINS recruits patients from 21 secondary healthcare sites across the UK (regionally being London, Sussex, Surrey, West Yorkshire, West Midlands, Kent, Bedfordshire, Lancashire). The study meets all ethical and consent standards set by local institutional review boards at each of the participating sites (Riverside Research Ethics Committee 04/Q0401/40). Detailed description of the BRAINS protocol has been published elsewhere [10]. The recruitment period was between 2014 and 2019. All cases admitted to BRAINS were reviewed by a pre-designated onsite neurologist/stroke physician, with the diagnosis of ischemic stroke confirmed with CT or MRI. Extensive demographic data including age, sex, and ethnicity was collected from a nurse-led interview as detailed in previously published protocols [10,11]. All adult (≥18 years old) participants at the time of ischemic stroke event were recruited. South Asians were identified by self-reporting as Indian, Pakistani, Sri Lankan or Bangladeshi [12].

### Ethics statement

Ethical approval was granted by the Riverside Research Ethics Committee (04/Q0401/40). Informed written consent was obtained from each case directly or via a surrogate in the presence of an onsite clinical research nurse.

### Clinical characteristics

AF cases were diagnosed with an ECG during the admission process or with a pre-existing diagnosis. Risk factors were defined as: hypertension diagnosed at discharge (≥140/90mmHg), previous diagnosis of hypertension or pre-stroke treatment with antihypertensive medication, hypercholesterolemia defined by previous diagnosis or serum cholesterol > 5.2 mmol/L, diabetes mellitus classified from a previous diagnosis of type I or II. Peripheral Vascular Disease (PVD) and ischemic heart disease data was collected from clinical records. Smoking and alcohol history were recorded if the patient currently/ever used. Obesity was defined by increased waist circumference (Men: >102cm, Women: >88cm) or BMI (≥30) [13]. Cardiovascular disease status was defined using previous/new diagnosis of PVD, ischemic heart disease (ischemic heart disease/angina, previous myocardial infarction), atrial fibrillation, previous ischaemic stroke, and TIA. Ischaemic stroke subtype was determined using the Trial of Org 10172 in Acute Stroke Treatment (TOAST) criteria by the onsite patient physician [14].

Medication history included treatment before admission and following discharge, including antiplatelet (aspirin, clopidogrel, dipyridamole) and anticoagulant (warfarin, rivaroxaban, apixaban, dabigatran, edoxaban) status. Anticoagulants were further subsets into Novel Oral Anticoagulants (NOAC) which included rivaroxaban, apixaban, dabigatran, edoxaban.

### Statistical analysis

Demographic details and categorical data between South Asians and white British patients were compared using a student t-test and prevalence of individual risk factors between ethnic groups and AF by χ^2^ test. A stepwise logistic regression was performed to identify association between AF status and ethnic groups adjusted for potential confounders. Variables were removed from the model if they crossed *P*>0.05 threshold. Variables considered to have an association with AF were selected with respect to literature and biological plausibility. Comparison of antiplatelet therapy depending on cardiovascular disease status used χ^2^ test. Little’s MCAR test used to assess for missing completely at random for multivariate data with missing values [15]. Treatment details were compared using χ^2^ test. All data used in this study was anonymised prior to analysis. A *P*<0.05 was taken as statistically significant.

## Results

### Atrial fibrillation risk

Of the 3515 individuals identified with ischemic stroke, 1482 (men:972, women:510) were South Asian and 2033 (men:1141, women:892) were white British. Of these, 190 (men:123, women:70) South Asians and 462 (men:252, women:210) white British stroke patients had confirmed AF (South Asians:13.0% vs white British:22.7%, *P*<0.001).

Demographic and clinical characteristics among AF status are presented in Table 1. Age of ischemic stroke onset was significantly earlier in non-AF cases (67.4 years, SD = 14.4) compared to AF cases (75.9 years, SD = 11.4) (mean difference = 8.5 years, *P*<0.001). Comparing ethnic differences for each AF status, average age of stroke was significantly lower in non-AF cases compared to new-AF cases for both South Asians (non-AF:64.1 years, SD = 14.8 vs new-AF:73.9 years, SD = 12.5, *P*<0.001) and white British stroke patients (non-AF:70.2 years, SD = 13.5 vs new-AF:77.5 years, SD = 9.8, *P*<0.001).Those with pre-existing AF have an older average age of onset compared to those without AF for both South Asians (non-AF: 64.1 years, SD = 14.8 vs pre-existing-AF: 72.7 years, SD = 12.3, *P*<0.001) and white British stroke patients (non-AF:70.2 years, SD = 13.5 vs pre-existing-AF:77.1 years, SD = 11.0, *P*<0.001). No significant difference in age of event is reported between new-AF and pre-existing AF (South Asian: *P* = 0.60, white British: *P* = 0.71). Among traditional AF risk factors, hypertension, and ischemic heart disease were significantly higher in patients with AF.

**Table 1:** Ischemic stroke population characteristics stratified by atrial fibrillation (AF) status.

	AF (n = 655)	Non-AF (n = 2860)	
	Mean (SD)	Mean (SD)	*P*-Value
**South Asian, *n* (%)**	193 (29.5)	1289 (45.1)	<0.001
**Age, years (SD)**	75.9 (11.4)	67.4 (14.4)	<0.001
**Men, *n* (%)**	375 (57.3)	1738 (60.8)	0.10
**TOAST Stroke Subtype**	***n*, (%)**	***n*, (%)**	***P*-Value**
**Large-artery atherosclerosis**	75 (14.4)	626 (28.6)	<0.001
**Small-vessel occlusion**	78 (15.0)	658 (30.0)	<0.001
**Cardioembolism**	303 (58.2)	200 (9.1)	<0.001
**Environmental Factors**	***n*, (%)**	***n*, (%)**	***P*-Value**
**Central obesity, *n* (%)**	151 (31.9)	720 (32.2)	0.89
**Smoking history, *n* (%)**	296 (45.5)	1338 (47.1)	0.47
**Alcohol history, *n* (%)**	204 (35.9)	863 (34.2)	0.47
**Comorbidities**	***n*, (%)**	***n*, (%)**	***P*-Value**
**Hypertension**	535 (82.3)	2019 (70.9)	<0.001
**Diabetes**	199 (30.7)	997 (34.9)	0.038
**Hypercholesterolemia**	278 (43.8)	1236 (43.8)	0.99
**Ischemic heart disease**	220 (36.2)	622 (23.3)	<0.001
**Peripheral vascular disease**	42 (7.1)	100 (3.8)	<0.001

n, sample size; Central obesity classified by waist circumference (men: >102cm, women: >88 cm) or BMI (≥30 kg/m^2^). Stroke subtype uses the TOAST (trial of ORG 10172 in acute stroke treatment) classification.

To estimate the possible confounding factors of the relationship of ethnic group and AF-status by demographic and clinical characteristics, comparisons between South Asian and white-British among those with AF are presented in Table 2. South Asians average age of ischemic stroke was significantly earlier (72.9 years,SD = 12.3) compared to white British patients (77.2 years,SD = 10.7) (mean difference = 4.4 years,P<0.001). South Asians patients were on average 3.9cm shorter and had greater prevalence of traditional AF risk factors compared to white British despite having lower incidence of AF (Table 2).

**Table 2:** Population characteristics with confirmed atrial fibrillation status stratified by ethnicity.

	South Asian (n = 193)	White British (n = 462)	
	Mean (SD)	Mean (SD)	*P*-Value
**Age, years**	72.9 (12.3)	77.2 (10.7)	<0.001
**Men, *n* (%)**	123 (63.7)	252 (54.5)	0.030
**TOAST Stroke Subtype**	***n*, (%)**	***n*, (%)**	***P*-Value**
**Large-artery atherosclerosis**	7 (4.5)	68 (18.6)	<0.001
**Small-vessel occlusion**	18 (11.5)	60 (16.4)	0.15
**Cardioembolism**	105 (67.3)	198 (54.2)	0.006
**Environmental Factors**	***n*, (%)**	***n*, (%)**	***P*-Value**
**Height, cm**	165.7 (9.9)	169.6 (9.7)	0.043
**Central obesity, *n* (%)**	49 (31.4)	102 (32.1)	0.88
**New diagnosis, *n* (%)**	32 (16.6)	99 (21.4)	0.16
**Smoking history, *n* (%)**	56 (29.2)	240 (52.4)	<0.001
**Alcohol history, *n* (%)**	41 (22.5)	163 (42.1)	<0.001
**Comorbidities**	***n*, (%)**	***n*, (%)**	***P*-Value**
**Hypertension**	174 (91.6)	361 (78.5)	<0.001
**Diabetes**	103 (53.9)	96 (21.0)	<0.001
**Hypercholesterolemia**	111 (61.0)	167 (36.9)	<0.001
**Ischemic heart disease**	101 (54.0)	119 (28.3)	<0.001
**Peripheral vascular disease**	7 (3.9)	35 (8.5)	0.049
**Treatment at admission**	***n*, (%)**	***n*, (%)**	***P*-Value**
**Anticoagulation**	55 (28.5)	130 (28.1)	0.93
**Antiplatelet**	91 (47.4)	125 (29.9)	<0.001
**Combined**	15 (7.8)	10 (2.4)	0.002
**Treatment at discharge**	***n*, (%)**	***n*, (%)**	***P*-Value**
**Anticoagulation**	87 (45.1)	199 (43.1)	0.64
**Antiplatelet**	94 (49.5)	143 (34.7)	0.001
**Combined**	34 (17.9)	33 (8.0)	<0.001

n, sample size; Central obesity classified by waist circumference (men: >102cm, women: >88 cm) or BMI (≥30 kg/m^2^). Stroke subtype uses the TOAST (trial of ORG 10172 in acute stroke treatment) classification.

To evaluate the association of AF status with the ethnic group among ischemic stroke cases, a logistic regression was performed. Unadjusted analyses showed South Asians had a lower OR of AF (OR:0.51,95%CI:0.43:0.61, *P*<0.001). We ran a stepwise logistic regression which included age, ischemic heart disease, hypertension, and smoking history as significant variables. Adjusted for these factors, the lower OR of AF in South Asians patients were maintained (OR:0.40, 95%CI:0.33:0.49, *P*<0.001). The associations of age of stroke event and other predictors with AF are reported in S1 Table.

### Treatment

Differences in antiplatelet and anticoagulant treatment among AF patients are presented in Table 2. South Asians with AF had significantly higher antiplatelet treatment on admission (South Asian: 47.4% vs white British: 29.9%, *P*<0.001) and discharge (South Asian: 49.5% vs. white British: 34.7%, *P* = 0.001). No significant difference was seen between anticoagulation treatment at admission (South Asian: 28.5% vs. white British: 28.1%, *P* = 0.93) or discharge (South Asian: 45.1% vs. white British: 43.1%, *P* = 0.64). Combination of treatment for admission and discharge is presented in S1 and S2 Figs.

**Fig 1:**
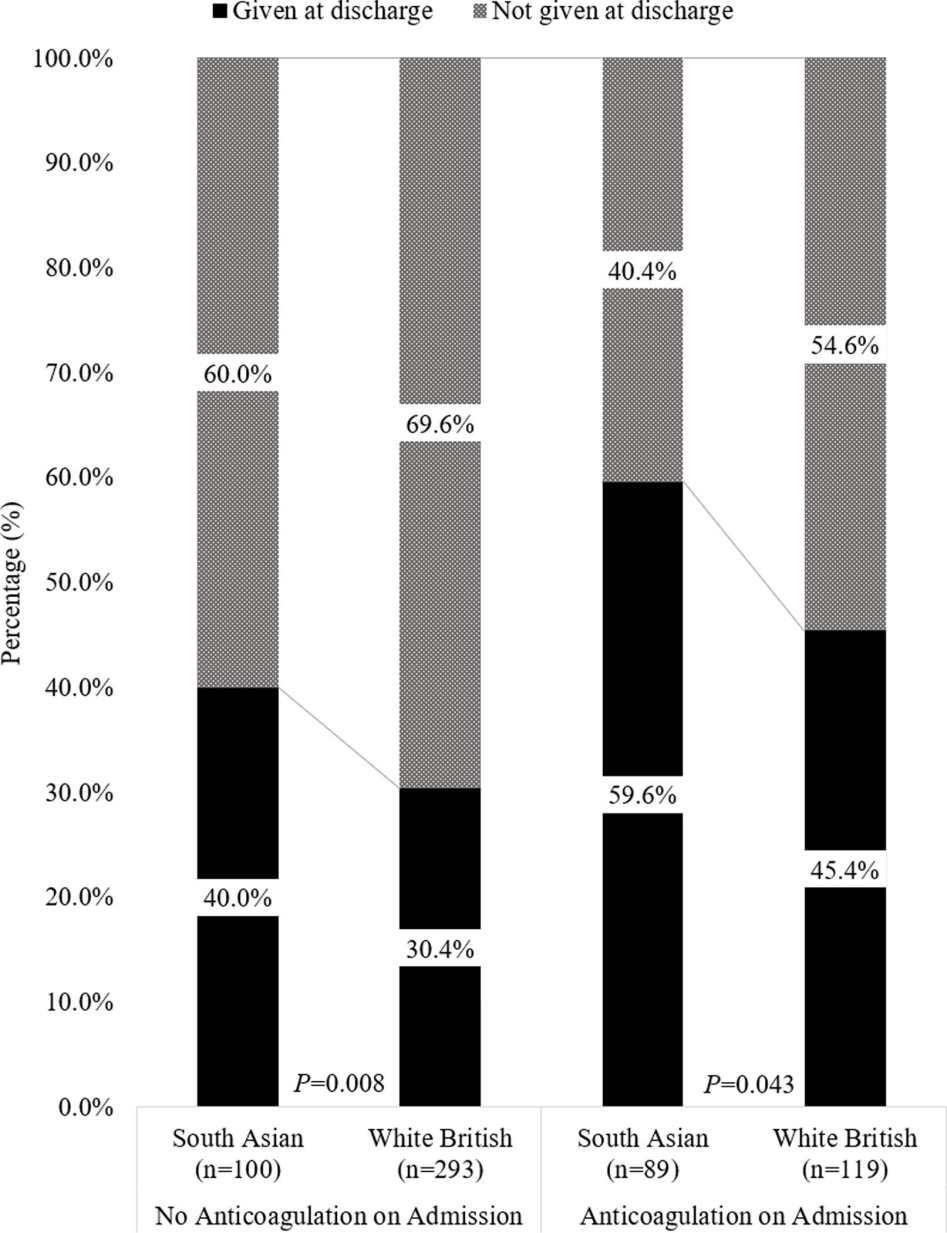
Changes in anticoagulation treatment from admission to discharge in confirmed AF cases, stratified by ethnicity. Legend: Chi-Square test used to compare between ethnicities. Anticoagulation treatment only.

Changes in anticoagulation status from admission to discharge are presented in Fig 1. Increased prevalence of antiplatelets therapy in South Asians was hypothesised to be due to increased risk of cardiovascular disease. To test this, we compared antiplatelet prevalence between South Asians and white British stroke patients. Of those with cardiovascular disease, 53.8% were South Asians and 49.2% were white British patients (*P* = 0.007). In those with cardiovascular disease, South Asians patients continued to show an increased prevalence of antiplatelet treatment at admission (South Asian: 69.1% vs. white British: 45.5%, *P*<0.001) and discharge (South Asian: 77.1% vs. white British: 60.7%, *P*<0.001). This difference was also present in those with AF at admission (South Asian: 47.4% vs. white British: 29.9%, *P*<0.001) and discharge (South Asian: 49.5% vs. white British: 34.7%, *P* = 0.001). In those without cardiovascular disease, South Asian patients continued to display a greater prevalence of antiplatelet therapy in the total ischaemic population only (Table 3).

**Table 3:** Antiplatelet treatment at admission and discharge stratified by ethnicity and cardiovascular disease.

	Antiplatelet Treatment	*n*, (%)	*n*, (%)	*P*-value
**Cardiovascular Disease**	**Total ischaemic population**	**South Asian (n = 794)**	**White British (n = 932)**	
	**Admission**	549 (69.1)	424 (45.5)	<0.001
	**Discharge**	604 (77.1)	559 (60.7)	<0.001
	**AF patients only**	**South Asian (n = 190)**	**White British (n = 412)**	
	**Admission**	91 (47.4)	125 (29.9)	<0.001
	**Discharge**	94 (49.5)	143 (34.7)	0.001
**Without Cardiovascular Disease**	**Total ischaemic population**	**South Asian (n = 682)**	**White British (n = 865)**	
	**Admission**	176 (25.8)	149 (17.2)	<0.001
	**Discharge**	589 (87.3)	720 (83.3)	0.032

Cardiovascular Disease (CVD) definition includes: PVD, ischemic heart disease (IHD, Angina, Previous MI), atrial fibrillation, previous ischaemic stroke, and TIA. n, sample size.

## Discussion

We show that South Asian ischemic stroke patients had a significantly lower prevalence of AF compared to their white British counterparts (13.0% vs. 22.7%, *P*<0.001). South Asians with AF presented with an ~4 year earlier stroke event compared to their white British counterparts and were more likely to receive antiplatelet therapy at both admission and discharge despite having similar cardiovascular profiles, but no significant difference was seen overall in anticoagulation rates.

The significantly lower AF prevalence in South Asians, despite higher prevalence of AF risk factors, shown here is consistent with findings from previous smaller studies [12,16] likely explained by morphologically smaller left atriums [17,18]. Though these phenomena have been recorded in South Asians, there are several potential underlying mechanisms such as differing atrial electrophysiological parameters. In this study we did not collect data on atrial morphology though we report South Asians being significantly smaller than white British with atrial fibrillation. Though height in our study was not independently associated with atrial fibrillation risk, this could be due to a strong association between ethnicity and height. Currently, no confirmed South Asian specific genetic polymorphism has been reported which could result in the protective effect seen [19].

Treatment with anticoagulant therapy at discharge remained relatively low, 45.1% in South Asians and 43.1% in white British. A previous study found 12.8% of ischemic stroke cases who were not being treated with anticoagulation treatment at admission were ineligible for treatment at discharge [20]. We also show rates of those untreated with AF prior to admission were relatively high in both ethnicities. This rate of treatment for white British cases is similar to those reported in previous studies, however no such data has been reported for UK residing South Asians [20]. One possible reason for relatively low treatment could be a result of poor awareness of the condition and the importance of treatment. A small UK study (n = 93) reported that only 49% of patients with AF could name the condition at baseline and 52% knew that anticoagulants prevent blood clots forming [21].

Antiplatelet only treatment was the most common treatment at admission for both ethnicities, though was greater in South Asians. A similar study has also reported a higher percentage of South Asians being treated with antiplatelet therapies with ischemic stroke, though this was not exclusive to AF cases [16]. Our study analysis extended to assess the treatments prescribed in those with AF. Combined treatment of antiplatelet and anticoagulant treatment was significantly higher both at admission and discharge in South Asians. Over the course of the lifetime of BRAINS, there was a transition from the use of Warfarin to NOAC’s. This preferred medication allowed more intervention eligibility for AF patients because of reduced bleeding risk associated with NOACs, and this benefit is disproportionately seen greater in South Asians [22,23]. However, even with that individual reduced bleeding risk, combined treatment with antiplatelets can increase the risk of major bleeding, including intracranial haemorrhage [24,25]. It is possible that the increased use of lone antiplatelets and/or combined with NOAC (also seen in a previous study [16]) is likely due to the increased prevalence of cardiovascular disease in South Asians. However, South Asians with cardiovascular disease were still more likely to receive antiplatelet treatment, regardless of AF status. Furthermore, in those without cardiovascular disease, South Asians continued to have a higher prevalence of antiplatelet therapy. We are not able to address individual management of cardiovascular disease, but a view may still exist that aspirin is beneficial for primary prevention providing a possible explanation for its use in those without cardiovascular disease.

### Limitations

BRAINS is an ongoing long-term study and during its lifetime treatment for AF has advanced from VKA (Warfarin) to non-VKA (NOAC). With this change, cases with AF who might not have been eligible for vitamin-K antagonists due to increased bleed risk could now be prescribed non-vitamin-K antagonists. This transition could result in a non-differential bias but as this study compares differences between two ethnic groups, this should not affect the overall result. Additionally, the transition from CHADS_2_ to CHA_2_DS_2_-VASc to calculate stroke risk could mean people who were assessed under the previous category may not have received anticoagulation [26]. While this transition could have resulted in a non-differential bias, we compared ethnic differences in the percentage of those prescribed with anticoagulants before and after June 2014, the date CHA_2_DS_2_-VASc guidelines were introduced into the NICE protocol [27]. Regardless of date, anticoagulation treatment prior to admission was not significantly different between ethnicity (CHADS_2_: *P* = 0.094, CHA_2_DS_2_-VASc: *P* = 0.206), and thus any effect of this transition between these protocols would be minimal. This study is not able to report adherence to anticoagulation in South Asians who may have poorer anticoagulation control due to socioeconomic factors [28] which could, in part, explain their 4-year lower stroke event age seen in this study. Furthermore, NICE recommends that patients do not receive anticoagulant treatment two weeks post stroke event [29]. Thus, patients may be re-prescribed anticoagulants either in hospital (if not discharged for 2 weeks) or post discharge. As BRAINS is a hospital study, we did not collect information on patients’ post discharge, we are unable to comment on whether patients received anticoagulation after the two-week period, potentially thus under reporting anticoagulant treatment prevalence.

Throughout the data collection process, new antiplatelet treatments have been offered. Of these Ticlopidine is not commonly prescribed to UK patients. Prasugrel and Ticagrelor were recommended for preventing atherothrombotic events by NICE in July 2014 mostly in cardiac disease and are not commonly used in ischaemic stroke.

Potential confounders may have varied over time, including awareness of stroke risk factors. Self-reporting of ethnicity and details of migration can increase the risk variability within this ethnic group [30]. Furthermore, we did not collect data on length of stay of South Asians in the UK. Ethnicity in this study was defined if patients’ grandparents originated from those regions. This data relied on self-reporting and thus we are unable to confirm the accuracy of their response. Defining ethnicity from a simple preselected list has a potential to result in ‘concealed heterogeneity’ [31]. However, previous studies have shown that such self-reported data is accurate for determining ethnicity [32,33]. We were unable to address socioeconomic data which is often used to explain a worse outcome as this was not in the original scope of BRAINS. Additionally, as BRAINS is a hospital-based study, we are unable to comment on community incidence of AF. To assess the representation of the UK residing South Asians within our study we utilised the broader BRAINS dataset, which consists of both ischaemic and haemorrhagic events and found similar stroke subtype prevalence across both groups in our study [12,34]. Our dataset reports 84.5% of ischaemic events in UK residing South Asians in BRAINS, similar to a previous reporting prevalence [12]. The UK recruitment sites were chosen to ensure a representative sample was recruited, being 21 hospital sites located in regions with high numbers of South Asians. We are unable to report specific effects of migration and how they develop as long-term follow-up was not undertaken.

## Summary

South Asians have a lower risk of atrial fibrillation compared to their white British counterparts. Though no significant difference was seen in use of anticoagulation at admission or discharge, use of antiplatelet medication alone in AF subjects was greater in South Asians.

## Supporting information

S1 Fig Distribution of anticoagulation and antiplatelet status in confirmed AF cases, stratified by ethnicity, at admission. Antiplatelet only and Anticoagulation only treatment included those were receiving only one of those treatments. Both Treatment included those receiving both antiplatelet and anticoagulant treatments. Chi-Square test used to compare between ethnicities.
(TIF)

S2 Fig Distribution of anticoagulation and antiplatelet status in confirmed AF cases, stratified by ethnicity, at discharge. Antiplatelet only and Anticoagulation only treatment included those were receiving only one of those treatments. Both Treatment included those receiving both antiplatelet and anticoagulant treatments. Chi-Square test used to compare between ethnicities.
(TIF)

S1 Table Associations of age of stroke event and other predictors with Atrial Fibrillation. Every model is adjusted by age and sex. Interaction model adjusted for age, sex, specific risk factor and an interaction variable. Central obesity classified by waist circumference (men: >102cm, women: >88 cm) or BMI (≥30 kg/m2).
(DOCX)

S2 Table STROBE statement—checklist of items that should be included in reports of observational studies. (DOCX)
