# Supplementary figures and images for: Atrial fibrillation in UK South Asian hospitalized ischemic stroke patients: The BRAINS study
Source: PLoS One. 2023 Feb 7;18(2):e0281014. doi: 10.1371/journal.pone.0281014 (PMC9904493; doi:10.1371/journal.pone.0281014)

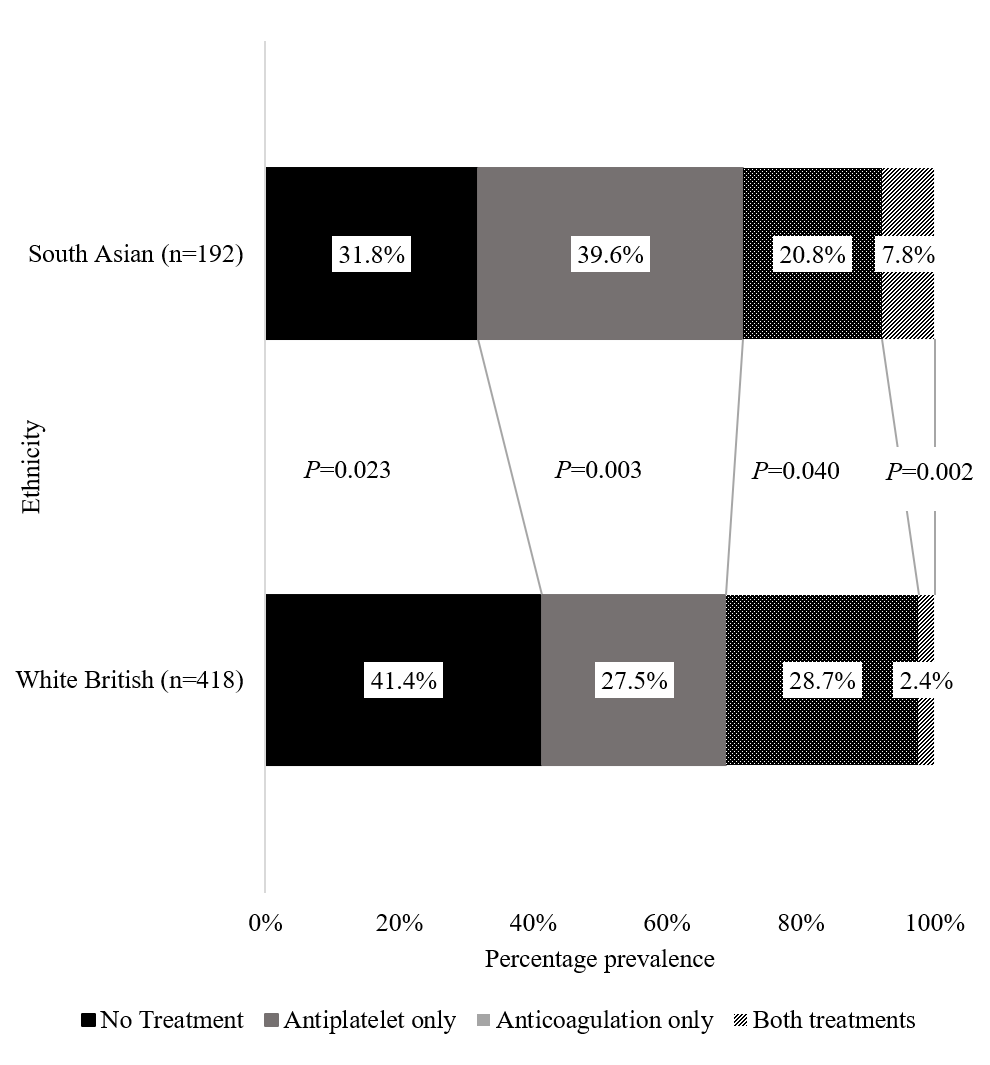

Supplement: S1 Fig — Antiplatelet only and Anticoagulation only treatment included those were receiving only one of those treatments. Both Treatment included those receiving both antiplatelet and anticoagulant treatments. Chi-Square test used to compare between ethnicities. (TIF) [file pone.0281014.s001.tif]

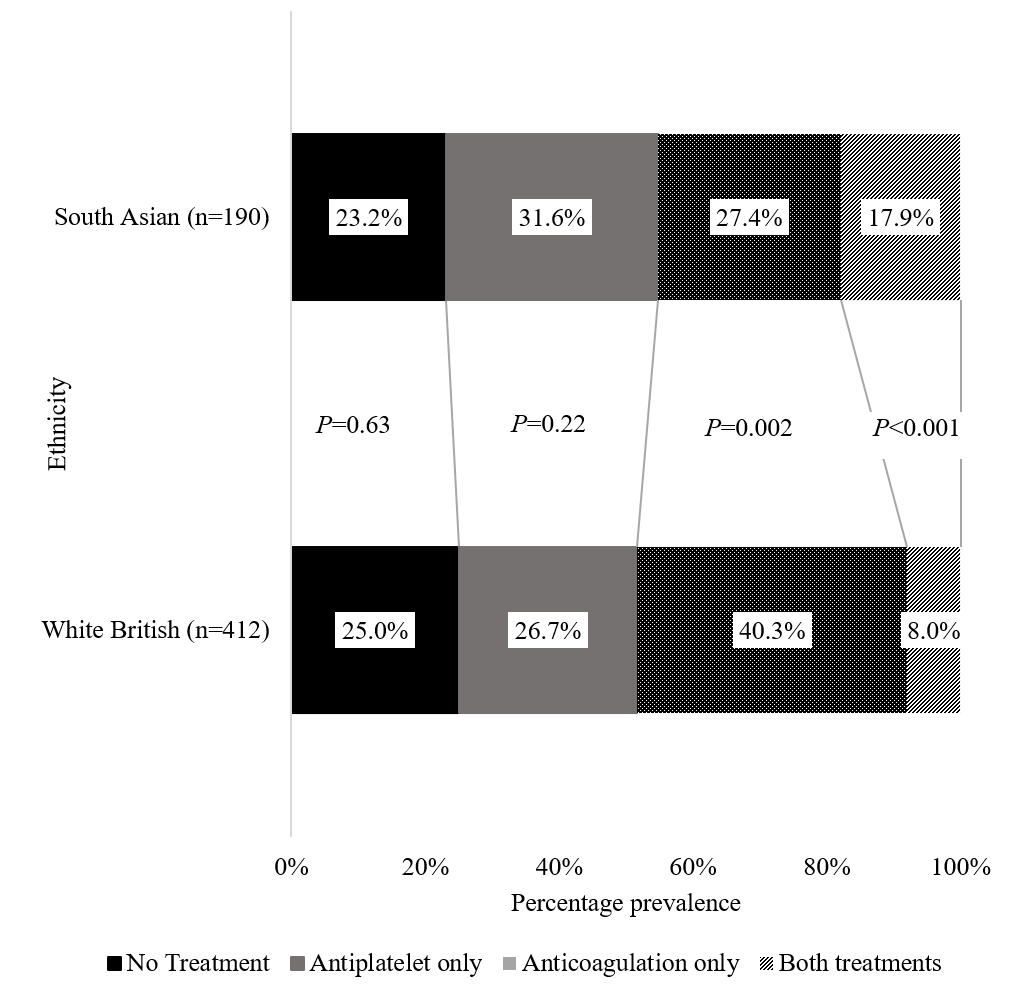

Supplement: S2 Fig — Antiplatelet only and Anticoagulation only treatment included those were receiving only one of those treatments. Both Treatment included those receiving both antiplatelet and anticoagulant treatments. Chi-Square test used to compare between ethnicities. (TIF) [file pone.0281014.s002.tif]
