# Supplementary material for: Atrial fibrillation in UK South Asian hospitalized ischemic stroke patients: The BRAINS study
Source: PLoS One. 2023 Feb 7;18(2):e0281014. doi: 10.1371/journal.pone.0281014 (PMC9904493; doi:10.1371/journal.pone.0281014)
Supplement: S1 Table — Every model is adjusted by age and sex. Interaction model adjusted for age, sex, specific risk factor and an interaction variable. Central obesity classified by waist circumference (men: >102cm, women: >88 cm) or BMI (≥30 kg/m2). (DOCX) [file pone.0281014.s003.docx]

**Table. S1** – **Associations of age of stroke event and other predictors with Atrial Fibrillation.**

| **Model (Age + sex)** | **Age** | | **Interaction** | |
| --- | --- | --- | --- | --- |
|  | **OR (95% CI)** | **P-value** | **OR (95% CI)** | **P-value** |
| **Hypertension** | 1.05 (1.04 – 1.06) | <0.001 | 1.06 (1.05 – 1.07) | <0.001 |
| **Diabetes** | 1.05 (1.04 – 1.06) | <0.001 | 1.05 (1.04 – 1.06) | <0.001 |
| **Hypercholesterolemia** | 1.05 (1.04 – 1.06) | <0.001 | 1.05 (1.04 – 1.06) | <0.001 |
| **Ischemic heart disease** | 1.05 (1.04 – 1.06) | <0.001 | 1.05 (1.04 – 1.06) | <0.001 |
| **Peripheral vascular disease** | 1.05 (1.04 – 1.06) | <0.001 | 1.05 (1.04 – 1.06) | <0.001 |
| **Central Obesity** | 1.05 (1.04 – 1.06) | <0.001 | 1.06 (1.05 – 1.07) | <0.001 |
| **Smoking** | 1.05 (1.04 – 1.06) | <0.001 | 1.04 (1.03 – 1.05) | <0.001 |
| **Alcohol use** | 1.05 (1.04 – 1.06) | <0.001 | 1.06 (1.05 – 1.07) | <0.001 |

Every model is adjusted by age and sex. Interaction model adjusted for age, sex, specific risk factor and an interaction variable. Central obesity classified by waist circumference (men: >102cm, women: >88 cm) or BMI (≥30 kg/m^2^).
